# Interpretation of Epidemiological Studies on the Relationship Between Mobile Phone Use and Cancer

**DOI:** 10.3390/epidemiologia7030086

**Published:** 2026-06-17

**Authors:** Michael Kundi, Hans-Peter Hutter

**Affiliations:** Department of Environmental Health, Center for Public Health, Medical University Vienna, Kinderspitalgasse 15, 1090 Vienna, Austria; hans-peter.hutter@meduniwien.ac.at

**Keywords:** brain neoplasms, epidemiology, risk factors, mobile phones

## Abstract

Background: In May 2011 the IARC (International Agency for Research on Cancer) classified radiofrequency electromagnetic fields as a possible human carcinogen mainly based on epidemiological studies about the association between mobile phone (MP) use and brain tumors. Considering that brain tumors have long latencies of around 30 years, it is unlikely that this association is due to an ‘initiating’ activity of MPs since virtually all studied brain tumor cases must have had already a covertly growing tumor when they started MP use. But there could be other adverse effects exerted by a MP when acting on later stages of malignant development. We propose that MP use acts adversely by increasing tumor growth rate and model it by an impact on the latency distribution shifting the age-incidence function to younger age. Methods: We calculate (1) relative risks (RRs) for MP use in comparison to the meta-analytic RR estimate for glioma in adults; (2) RRs for neuroepithelial childhood brain tumors in comparison to the findings of the MOBIkids study; and (3) hazard ratios in comparison to the results of the Million Women Study (MWS). Results: The meta-analytical odds ratio for glioma and long-term MP use in adults of 1.22 (95% confidence-interval: 1.02–1.46) could be explained by a shift in the age-incidence function by 32% of MP usage duration. Applying a 20% shift for childhood neuroepithelial brain tumors reproduced the ORs that were predominantly less than 1 in the MOBIkids study. For glioma risk in perimenopausal women in relation to long-term MP use in the MWS we found hazard-ratios close to 1 applying a 32% shift in the age-incidence function. Conclusions: The standard interpretation of relative risk estimates must be revised if exposure to the agent commenced after the malignant development has already started. All reported RR estimates of MP use can be reproduced by positing MP use increased tumor growth rate. However, since these results are obtained applying a modeling approach, further tests using epidemiological methods, which will be difficult or hardly feasible, or utilizing more promising laboratory methods are needed.

## 1. Introduction

Carcinogenesis is thought to be a multistage process with genetic and epigenetic cellular transformations being key elements driving progression through the stages. Despite difficulties in discriminating some of these stages, initiation, promotion, progression, invasion, and regional and distant metastases formation are considered important phases of this process [1,2]. A carcinogen can affect any of these phases and it is considered to take, in general, many years from exposure to a carcinogenic agent until clinical signs develop and diagnosis is established. For carcinogens that are in principle able to induce cancer, it is convenient to discriminate between the induction and the latency periods [3]. The induction period refers to the time from onset of exposure until malignant conversion occurs, while the latency period reflects the time from reaching this stage until clinical manifestation or detection. Both periods are considered to take many years. However, in practice it is typically impossible to distinguish between induction and latency periods and in the scientific literature it became common practice to combine them and refer to the total length as “latency” [4].

Studies investigating latency for cancer are limited, e.g., concerning ovarian cancer latencies of 30 to 40 years have been suggested [5], for colon cancer 10 to 25 years [6]. Similar latency periods were suggested for brain tumors [7,8,9,10]. Applying a Weibull model, Nadler and Zurbenko estimated latencies for 44 cancer types and found values between 7 and 57 years [11].

Often a distinction is made between agents that act during initiation and those acting during latent tumor development. Theoretically, such a distinction makes sense as an agent acting by increasing the probability of malignant transformation (e.g., because of its genotoxicity) will be associated with longer latencies as an agent that acts during latent growth (e.g., by increasing growth rate). Such agents that act during latent growth are often termed promoters. However, in most cases such a distinction is practically without value because we do not know at which stage an agent has acted. Since also progression through the stages is induced by genetic events, a differentiation referring to the genotoxicity of the agent in questions does not help either [12].

Concerning a possible association between mobile phone (MP) use and brain tumors, the latency of these neoplasms is crucial for the interpretation. Although the development of MP technology dates back to the 1950s, a relevant proportion of the population started MP use only after digital phones were marketed (between 1993 and 1995 in most countries). In some countries a low percentage of the population already had used the earlier analog technology to some extent, either as car or bag phones that are not associated with relevant exposure of the head region, and only from about 1987 onwards were handheld phones available [13]. Hence, in almost all case–control studies that recruited their subjects at most until 2004 [14,15,16,17,18,19,20,21,22,23], the longest latencies (in the sense of the duration between start of MP use and diagnosis) were 17 years but most were much shorter and hence lower than the average latency (the mean of the distribution of latencies from tinea capitis x-ray treatment studies: Sadetzki et al. [9]—28 years, atomic bomb survivors: Brenner et al. [24]—38 years; or from population incidence estimates: Nadler and Zurbenko [11]—22 years). Shorter latencies have been reported for brain tumors as secondary malignancies after treatment of a primary neoplasm although with a wide range (D’Elia et al. [25]—1–36 years for low-grade glioma; Walter et al. [26]—median latency for high-grade gliomas 9.1 years). From this it follows that in almost all primary brain tumor cases that had used a MP, usage started after the tumor was already initiated. In studies with cases recruited up to 2009 [27,28,29,30], the proportion of cases with latencies reaching or exceeding 10 years is higher, but still in almost all of them usage will have commenced during an already ongoing tumor development.

**Figure 1:**
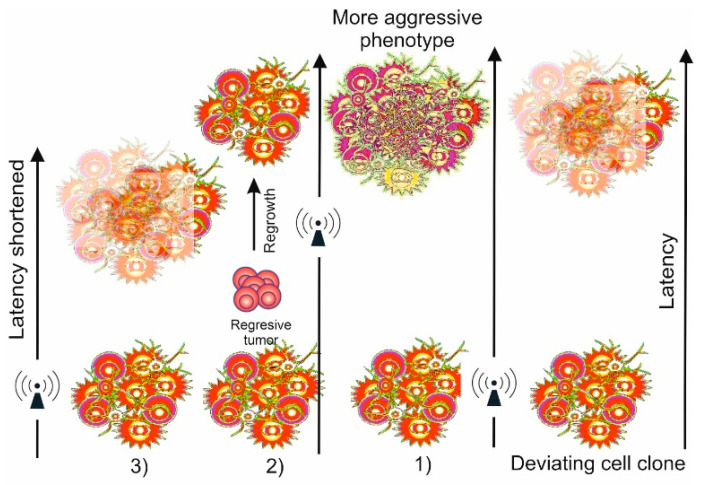
Theoretically, there are three adverse effects that an agent such as the radiation from a mobile phone can have on an already existing deviating cell clone that will lead to a diagnosis of cancer after a certain latency in an unexposed individual (rightmost scheme): 1) it can lead to a more aggressive tumor type (shift in tumor grade); 2) regrowth of a dormant or regressive tumor; or 3) in faster growth of the tumor and, consequently, shorter latency.

Until now, epidemiology has paid comparatively little attention to the analytical consequences of studying risk factors that act during development of a malignancy. Theoretically, there are three adverse effects an exposure to a risk factor can have if it commences during covert development of a neoplasm after it has surpassed the stage of a pre-malignant lesion: (1) it may lead to further genomic alterations that induce progression to higher malignant stages; (2) it may induce growth of a dormant or regressive tumor; and (3) it may increase tumor growth rate (Figure 1). Only the second possibility would actually increase incidence, because a dormant or regressive tumor may never become diagnosed in the lifetime of the individual without the exposure. The first possibility has no influence on incidence but shifts the distribution of diagnostic entities towards higher grades only. The third one has an impact on incidences too, but not in the sense of an increased incidence in general (especially not in childhood and adolescence when competing causes of death are rare), but in the sense of a shift in the age-incidence function to younger age because faster tumor growth leads to earlier diagnosis.

Many epidemiological studies of risk factors of cancer will face the problem of an uncertain temporal relationship between exposure and latent tumor development. Many of these factors may act on individuals in whom the tumor is already present. Only few agents will actually act before a neoplastic development has already started. We do not here refer to a distinction between initiating and promoting agents, as this distinction is maybe of little value [12], but to the natural history of chronic diseases such as cancer that have latencies of decades. Both cohort as well as case–control studies may only include a small fraction of cases in whom malignant development started after onset of the exposure in question. One example, elaborated in detail in Supplement 1, is hormone replacement therapy and breast cancer [31,32,33,34,35]. Another one is oral contraceptives use and breast cancer risk [36].

It has to be borne in mind that latencies are not the same neither for all malignancies nor for any selected cancer type for all individuals. Latencies have a distribution within the population of those having undergone malignant transformation. This distribution is unknown; however, earlier literature has suggested it is of log-normal type [37]. This assumption would indicate that a large proportion of cases has latencies below the average but some have very long ones. A more general approach would utilize the Weibull distribution that can take on various shapes including those resembling a log-normal distribution [11].

If by the action of an agent, such as MPs, latency is reduced in the population of those exposed, the age-incidence function is shifted to younger age because diagnosis will be earlier. It has been suggested that if MPs act by promoting brain tumor growth, this effect may be detected even after a short usage duration [38]. In fact, as a tumor-growth-promoting agent can impact any stage after malignant transformation occurred, this assumption is not unfounded. However, as already shown in 2004 [39], the magnitude of the risk resulting from such a promoting action that can be determined in case–control or cohort studies depends on the shape of the age-incidence function. For brain tumors in the adult age range (from 20 to 70 years) this function is, in contrast to many other cancer types, characterized by a linear relationship between age and log-incidence. The slope of this linear function is very shallow (in the range of 0.03 to 0.05 for natural log) resulting in an equally small risk estimate for short durations of use and consequently small shifts in this function. The relative risk that can be estimated in a case–control or cohort study is approximated by the formula exp(ß*s), where *ß* is the slope of the age-log-incidence function and *s* is the shift from the promoting effect [40]. Taking the higher value, 0.05, of the range of slopes, in order to observe a doubling of the risk, the shift in the function must be approximately 14 years and duration of use must exceed this value. Hence, it is virtually impossible to detect an effect on brain tumor risk for small durations of use. Therefore, the IARC panel that assessed the available evidence on the relationship between MP use and brain tumors dismissed early studies with insufficient usage duration [41].

### 1.1. Special Considerations About Childhood Brain Tumors

The etiology of childhood and adult cancers underlies different mechanisms. Adult cancer is the consequence of progressive acquisition of genomic alterations and, hence, a consequence of aging and the accumulating lesions occurring spontaneously or from exposures to genotoxic agents. While adult cancers arise predominantly from epithelial tissues, such cancers are uncommon in children. Consequently, distribution of cancer types is vastly different between children and adults. Central nervous system (CNS) tumors comprise 20% of all childhood but only less than 2% of adult cancers [42,43,44]. Hematopoietic neoplasms are the most frequent malignancies below 20 years of age (43% of all cancers, Ferlay et al. [45]) but in adults they account for only 6–7%.

**Figure 2:**
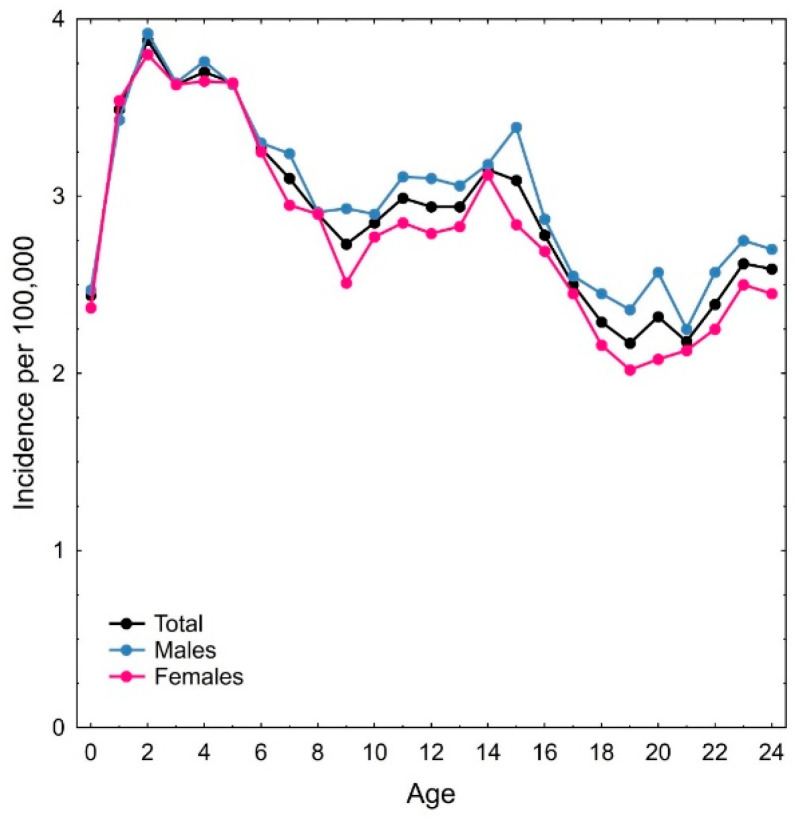
Annual incidence by gender and one-year age groups 2012–2016 of neuroepithelial brain tumors. Data from the Central Brain Tumor Registry of the United States (CBTRUS, special analysis, courtesy of Carol Kruchko).

CNS cancers in children are due to an increased susceptibility of neural tissues for malignant transformation during embryonic and/or fetal development. Indeed, most if not all pediatric CNS cancers are initiated during pregnancy while the neural tube and the CNS is being built. The different types of medulloblastoma, which are the most frequent CNS tumors in children, originate from fetal precursors of the cerebellum and dorsal brain stem [46,47]. For high-grade childhood glioma, fetal progenitors have also been demonstrated [48,49]. Some CNS tumors are already diagnosed at birth (especially retinoblastoma and atypical teratoid rhabdoid tumors) [50]. These facts are strongly supporting the assumption that neural cells or progenitors are exposed to the risk of malignant transformation during windows of susceptibility in certain periods of embryonic and fetal development where they may be prone to initiate tumorigenesis after specific genomic alterations [51]. Consequently, the incidence of CNS tumors, and in particular of neuroepithelial brain tumors, is, after an initial rise, decreasing from early years of life to adulthood (Figure 2).

### 1.2. Risk Indicators and Their Relationship to the Age-Incidence Function

Because adult cancer is predominantly a consequence of aging, incidence is typically increasing with age, leading to a positive slope of the age-incidence function with some cancers showing a decline at advanced age that is maybe due to competing causes of death. If exposure to an agent results in increased tumor growth rates, the earlier diagnosis and consequently the shift in the age-incidence function to younger age will result in odds ratios and rate ratios (or hazard ratios) above 1 if this function has a positive slope. However, under the premise of an increased tumor growth rate due to the agent, inevitably these risk indicators will be less than 1, if the slope is negative.

If an agent acts upon an already growing tumor, it may induce regrowth of a regressing tumor and thereby lead to an increased incidence. However, such events might only occur infrequently and we hypothesize that an agent will predominantly be adverse by increasing tumor growth rate. The consequence will be that the tumor reaches the size earlier that is on average required for clinical symptoms and diagnosis to occur.

Consider a cohort of individuals that are exposed from a certain age onwards to a tumor-growth-promoting agent; then, compared to the unexposed, this cohort will have an age-incidence function that is progressively shifted to younger age as duration of exposure increases. If we let *I_a_* denote the incidence rate at age *a* in the unexposed cohort, then after onset of exposure at age *A*, incidence rate at age *a* > *A* in the exposed cohort will be equal to the incidence rate in the unexposed at some later age *a* + *x* (*x* > 0). The rate ratio will be *I_a_*_+*x*_/*I_a_* and therefore >1 if the slope is positive and <1 if the slope is negative. The same holds for the odds ratio in a case–control study for each age *a* at diagnosis and age *A < a* at onset of exposure.

In the following sections we will address three questions: (1) can the results on MP use and brain cancer from epidemiological studies in adult populations be explained by an effect on tumor growth? (2) how can the results of the MOBIkids study be interpreted in the light of the hypothesis of an effect of MP use on tumor development? and (3) can the results on MP use and brain cancer risk of hazard ratios close to 1 in peri-menopausal women reported in the Million Women Study be interpreted as a lack of adverse effects of this technology?

### 1.3. Question 1: Can the Results on MP Use and Brain Cancer from Epidemiological Studies in Adult Populations Be Explained by an Effect on Tumor Growth?

Referring to the discussion above on duration of use of a MP, we will focus here only on studies including a relevant number of long-term users. Furthermore, we will address the potential risk this use may exert on gliomas, because this aspect has been studied most frequently. As there are multiple data overlaps in the published literature, we will, if available, use the pooled results instead of the individual findings. Furthermore, we will only include case–control studies because the published cohort studies cannot contribute to risk assessment (the Million Women Study is discussed below, the Danish cohort study has severe limitations [52] and also the COSMOS trial [53] is uninformative regarding a possible risk [54]). This is not to say that case–control studies are without biases; however, the impact of potential biases on the results has been thoroughly studied (a discussion is provided in Supplement 2 referring to [55,56,57,58,59,60,61]).

We will meta-analytically combine this evidence to obtain an estimate of the relative risk in adults. Further, in a next step we will show that assuming an impact of MP use on tumor growth leading to earlier diagnosis is compatible with this risk estimate.

### 1.4. Question 2: What Are the Consequences for the Interpretation of Risk Estimates in the MOBIkids Study?

The MOBIkids study [62] is the largest case–control study in children, adolescents, and young adults on brain tumor risk and wireless phone use conducted so far. Main analysis focused on neuroepithelial tumors [63]. Brain tumors and especially those diagnosed at young age are a heterogeneous group of tumors that have different origins, different genetic and epigenetic features, differences in clinical appearance, in grade of malignancy and in the age of appearance. Therefore, a proper analysis has to consider these differences.

### 1.5. Question 3: What Are the Consequences for the Interpretation of the Million Women Study?

During 1996–2001, 1.3 million women born in 1935–1950 were participating in the UK National Health Service Breast Screening Programme and completed a postal questionnaire including questions about MP use around 2001. Of these women, 776,156 were followed up until end of 2017 by record linkage to the UK National Health Service Central Register with an average follow up of 14.2 years [64]. Participants were asked about 10 years later (around 2011) to provide information on their current and prior MP use, but these data have not been used in the published main analysis.

While such a cohort approach might be useful for some cancer types with a high incidence gradient during the period following 50–70 years of age at enrollment, this is not the case for malignant brain tumors. For those older than 65 years (around 10% of the cohort), the incidence even declines during part of the follow up (see Section 2.3). Consequently, the interpretation must consider these features of the age-incidence relationship.

## 2. Materials and Methods

### 2.1. MP Use and Brain Cancer from Epidemiological Studies in Adult Populations

We performed a meta-analysis of all case–control studies that reported odds-ratios for long-term MP users (>7 years) and gliomas using the DerSimonian–Laird random effect model. We used only pooled results in case individual studies were published with overlapping of the pooled data. Overall, four studies were included (Table 1). For illustrative purposes we show in the Forest plot results for analog and digital MPs separately [65] and also the results reported in Appendix 2 of the Interphone study [22]. These results did not enter the meta-analysis.

In order to analyze the age-incidence function and to determine the effect of a shift in this function on the relative risk in adults we downloaded US population-based data for 1992–2000, from 12 registries in the Surveillance, Epidemiology, and End Results (SEER) database (Atlanta, Detroit, Los Angeles, San Francisco, San Jose-Monterey, Seattle, rural Georgia, Connecticut, Hawaii, Iowa, New Mexico, and Utah). Since this database contains individual cases, incidence could be computed for each year of age from 20 to 84 years and for 85+ years. We included cases diagnosed with glioma (ICD-3 morphology codes 9380-9480) in non-Hispanic white males and females.

**Figure 3:**
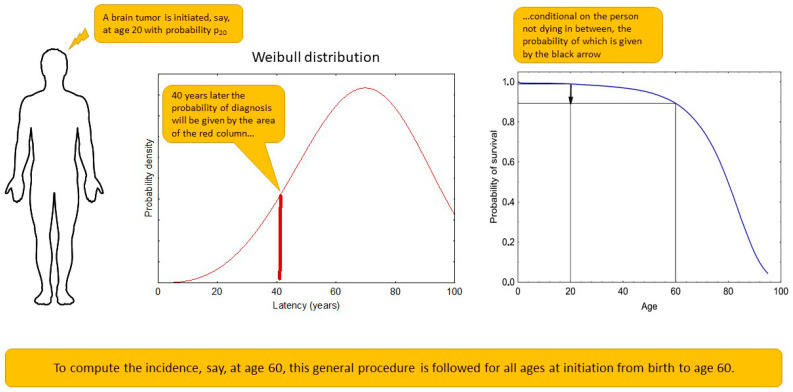
Illustration of the procedure to compute the age-incidence function.

In order to compute an expected shift in the age-incidence function from a tumor-growth-promoting agent, this function must be modeled first by considering tumor induction probability depending on age and the latency distribution as well as the probability of surviving to an age when the diagnosis would occur. A diagram (Figure 3) illustrates the principle and Equation (1) shows the translation into mathematical terms.

The incidences were fit using the following function (Equation (1)) with parameters estimated by the Levenberg–Marquardt algorithm. In the results section the incidences and the function fit for males and females combined are shown and in Supplement 2 (Figure S2.3) it is shown separately for males and females.

(1) $$I_{a}=\sum_{t=0}^{a}p_{t}\left(e^{-{\left(\frac{a-t}{T\cdot f(t)}\right)}^{b\cdot g(t)}}-e^{-{\left(\frac{a-t+1}{T\cdot f(t)}\right)}^{b\cdot g(t)}}\right)\left(1-S_{t}+S_{a}\right)$$

with *I_a_* denoting the incidence rate at age *a*. *p_t_* is the probability of malignant transformation at age *t*, parametrized as given in Equation (2). The term next to *p_t_* in brackets is the Weibull probability that the tumor will be diagnosed at age *a* if malignant transformation occurs at age *t*. The Weibull distribution is modeled with parameters *T* and *b* and predefined functions *f(t)* and *g(t)* allowing for an age dependency of the latency distribution (see Supplement 2, Table S2.2). The Weibull distribution has two parameters with *b* called the shape and *T* called the scale parameter. It has the cumulative distribution function (CDF) 1 − exp(−(*x*/*T*)*^b^*). Hence, if the latency has a Weibull distribution with parameters *b* and *T*, then the probability the diagnosis occurs in the year *x* is given by CDF(*x* + 1) − CDF(*x*). The final term in Equation (1) is the probability to survive from age *t* to age *a*.

(2) $$p_{t}=\frac{\pi_{t}}{1+e^{-c}}$$

We assumed that the probability of malignant transformation is not constant but is higher at (before) birth and declines thereafter. The obtained probabilities are shown in Supplement 2 (Table S2.2). Parameter *c* is estimated by the Levenberg–Marquardt algorithm and is the logit of the basic probability of transformation. Function *π_t_* was implemented to be ~1 at birth and then strongly declines until it reaches an almost constant level at age 40.

Since gliomas are a heterogeneous group of malignancies, an analysis based on histo- and molecular pathologically related entities could, in principle, be applied to provide a more specific modeling approach (e.g., with type specific latency distributions). However, since such data are not consistently reported in the studies included here, we cannot pursue this aspect further.

### 2.2. MP Use and Neuroepthelial Brain Tumors in the MOBIkids Study

For the purpose of interpreting the findings of the MOBIkids study, it is important to distinguish between the types of neuroepithelial tumors according to their age-incidence relationship. Pilocytic astrocytoma, the most frequent childhood glioma, shows a bimodal incidence function with a peak at 2 to 4 years of age and a second one at 12 to 14 years maybe due to different latencies of pilocytic subtypes. Malignant glioma NOS (not otherwise specified; not including glioblastoma) show a peak incidence at 4 years of age and decline thereafter. Ependymal and choroid plexus tumors peak in the first life years and then strongly decline. Neuronal and mixed neuronal–glial tumors have a peak at 15 years of age. Glioblastoma and oligodendroglioma increase in incidence during childhood and adolescence. All other neuroepithelial brain tumors remain virtually constant during childhood and adolescence and slightly increase in incidence only from 20 years onwards (Supplement 2, Figure S2.4).

As for adults (Equation (1)), we modeled incidences by assuming there is a certain probability of malignant transformation and that latencies follow a Weibull distribution and hence define the probability that the transformed cell clone will grow to be diagnosed at age *a*. In contrast to adults in whom malignant transformation may occur at any age, we assumed that childhood tumors are due to malignant transformation occurring already prenatally such that we have only one probability of malignant transformation. However, we fit the model to different tumor types (i) that were then summed up to give the total incidence at age *a*. Equation (3) shows these functions. We waived the survival term appearing in Equation (1) since in the age range 0 to 24 years it remains virtually 1 (dropping at most to 0.983 at age 24).

(3) $$I_{a}^{\left(i\right)}=p_{\left(i\right)}\left(e^{-{\left(\frac{a}{T(i)}\right)}^{b(i)}}-e^{-{\left(\frac{a+1}{T(i)}\right)}^{b(i)}}\right)$$

where *I_a_*^(*i*)^ is the incidence rate at age *a* of tumor type (i), *p*_(*i*)_ the corresponding probability of transformation (before birth) that will result in a tumor later in life. We modeled latency as a Weibull distributed variable with parameters *T* and *b* that were allowed to vary with tumor type (i).

In order to estimate the effect of MP use arbitrarily assuming a shift in the age-incidence function by 20% of the usage duration, we obtained the distribution of MP use by age groups (10–14, 15–19, and 20–24 years) from the MOBIkids study coordinators (Supplement 2, Figure S2.5).

### 2.3. MP Use and Malignant Brain Tumors in the Million Women Study

In order to investigate the expected outcome of a cohort study such as the Million Women Study in peri-menopausal women under the assumption that the gliomas studied were already covertly present at the time of enrollment and start to follow up, a different approach has to be chosen as compared to the methods described above for adult und childhood case–control studies. Women were started for follow-up during 1999 to 2005 and for each enrolled women MP use started at a certain year at or before this year or has not yet commenced. These differences in the composition of the cohort make it necessary to study the expected cumulative proportion without glioma diagnosis during the up to 18 years of follow-up taking the staggered entry into the study into account.

To estimate the impact of mobile phone use that acts upon an already initiated and growing neoplasm, the year of start of use in participants of the Million Women Study was estimated based on the data reported in Table 2 of Schüz et al. [64]. The distribution of birth years within the range 1935 to 1950 was estimated based on the mean and standard deviation (59.2 ± 4.6) and the proportion of those answering the questionnaire in each of the years 1999 to 2005 from the statement that the median was 2001 and the interquartile range 2000 to 2003.

**Figure 4:**
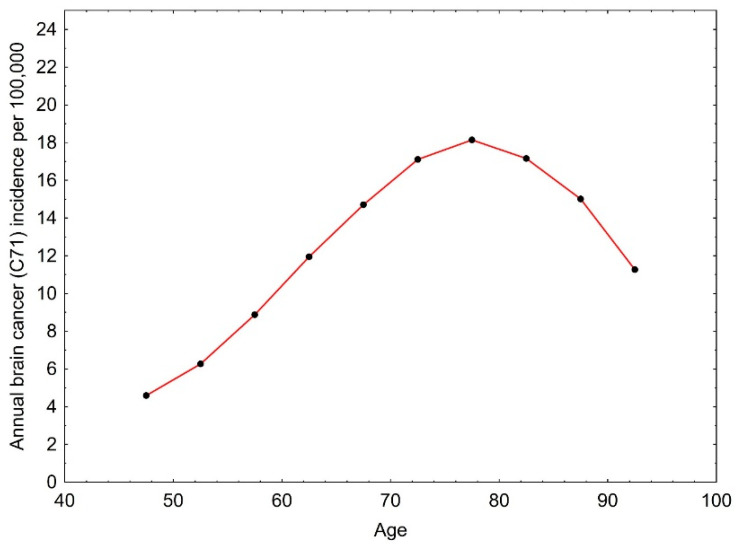
Annual incidence of malignant brain tumors (C71) in the UK averaged over 1999 to 2017 in the age range 45 to 95 in females. The points are placed in the middle of the 5-year age intervals as reported by the National Cancer Registration and Analysis Service (NCRAS).

Glioma incidences for women were obtained from the Office of National Statistics for England 1999 to 2017 (now the National Cancer Registration and Analysis Service (NCRAS) [67]). The incidences averaged over this time period are shown in Figure 4.

The estimate of the incidence in each year of follow up in the Million Women Study cohort was based on the assumption that in MP users the incidence expected for age *A* is shifted by 32% of the duration of MP use (the value obtained from case–control studies).

(4) $$\begin{array}{c} I_{t,u}=\sum_{y=1999}^{2005}\sum_{b=1935}^{1950}p_{y}p_{b}\sum_{s=1990}^{y}f_{s,b}^{*}I_{y+t-b+0.32(y+t-s)}S_{y-b,t}\\ I_{t,n}=\sum_{y=1999}^{2005}\sum_{b=1935}^{1950}p_{y}p_{b}\left[\sum_{s=y+1}^{min(y+t,2017)}f_{s,b}I_{y+t-b+0.32(y+t-s)}+(1-\sum f_{s,b})I_{y+t-b}\right]S_{y-b,t} \end{array}$$

Equation (4) shows the incidence estimates after *t* years of follow up in users (*u*) and non-users (*n*). *p_y_* and *p_b_* are the proportions of participants answering the questionnaire in year *y* and born in year *b*, respectively. *f_s_*_,*b*_ is the proportion of participants estimated to start mobile phone use in calendar year*s* if they are born in year *b*. Since users are defined as those starting use at the time or before they answered the questionnaire in 1999 to 2005, the proportions f* are summing up to 1 for each birth year *b* and year of inclusion *y*. Since the number of women dying during follow up is not negligible, the survival fraction *S_A_*_,*t*_ *t* years after reaching age *A* was included based on the life table for England from 2011 [68]. The estimates were then related to the number of women using and not using a MP in the Million Women Study for follow up until 2017. Time to diagnosis from start of follow-up was computed from the cumulative incidence function for MP users and non-users, a Cox regression was performed with MP use as factor, and time to brain cancer diagnosis as an outcome as has been done in the Million Women Study.

## 3. Results

### 3.1. MP Use and Brain Cancer from Epidemiological Studies in Adult Populations

**Figure 5:**
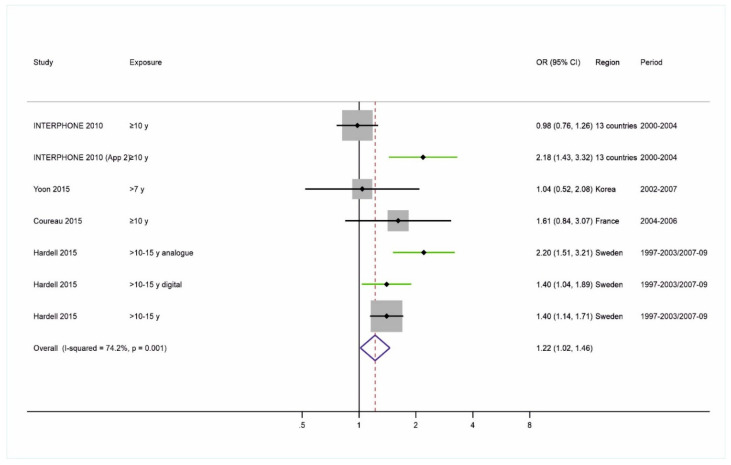
DerSimonian and Laird random effects meta-analysis of case–control studies of long-term mobile phone use and glioma risk. Note that results for digital and analog phones of the Hardell study and of Appendix 2 [22] of the INTERPHONE study are shown for completeness (emphasized by green color) and did not enter meta-analysis. Size of boxes proportional to weight, horizontal lines indicate 95% confidence intervals, diamond shape refers to the meta-analytical estimate with its wide indicating its 95% confidence interval.

The Forest plot for the studies with long-term MP use and glioma risk is shown in Figure 5. The pooled risk estimate was statistically significantly elevated with an odds ratio of 1.22 (95% confidence interval (CI): 1.02–1.46). Although the confidence intervals of all results included in the meta-analysis do overlap with the meta-analytic estimate, there was significant heterogeneity (I^2^ = 74.2%, *p* = 0.001). This heterogeneity can completely be removed if a correction for selection bias [61] is introduced. A meta-regression including an estimate of the magnitude of the selection bias (i.e., the logarithm of the selection odds ratio) led to an I^2^ close to zero. There are other biases that may have affected the outcomes as has been discussed previously [56,59,61,69], but since the current analysis is not focusing on an improved risk estimation, we provide a discussion in Supplement 2.

**Figure 6:**
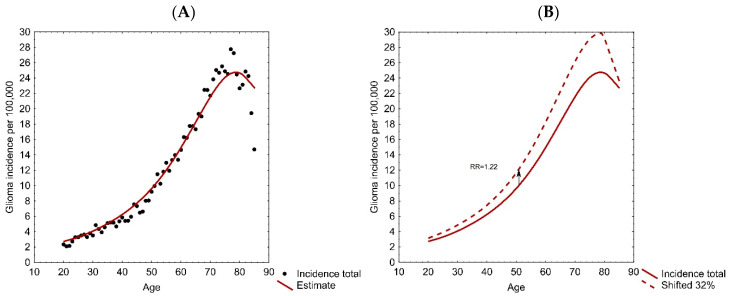
(**A**) Glioma incidence by age 29 to 85 from the SEER database (1992–2000) and fitted functions of type Equation (1) (**B**). Estimated incidence function and function shifted by 32% of the average MP usage duration that led to an average incidence ratio equal to the meta-analytical relative risk estimate of 1.22. The arrow indicates the rate ratio of 1.22 at age 50 (which lies in the middle of the age range covered by the case–control studies) and is at the same time the geometric mean of the rate ratios from 20 to 70 years.

In order to determine whether the obtained glioma risk is consistent with the assumption of a shift in the age-incidence function we fit the model shown in Equation (1) to the SEER data. The fit was excellent with pseudo R^2^ of 0.97 for males and 0.94 for females and 0.97 for the total (Figure 6A).

The average duration of MP use in the studies combined in the meta-analysis, estimated from the reported data in controls, is ~13 years. In order to determine whether the hypothesis of an effect on growth rate is compatible with the estimated odds ratio of 1.22, the age-incidence function for males and females estimated based on the SEER database was shifted beginning with 20% of the average duration of use and increased by 2% until the ratio at the shifted to the original incidence reached 1.22. This was the case at a shift of 32% (Figure 6B). Results for each step are shown in Supplement 2 Table S2.3.

### 3.2. MP Use and Neuroepthelial Brain Tumors in the MOBIkids Study

As shown in Table 2, odds ratios for different categories of MP use with respect to neuroepithelial brain tumors were numerically below 1 and only in the oldest age group (20 to 24 years) risk estimates were slightly above 1. However, the interpretation that “Overall, our study provides no evidence of a causal association between wireless phone use and brain tumours in young people” [62] driven by the failure to detect significantly increased odds ratios neglects the fact that given the falling trend of brain tumor incidences and the natural history of the disease, within the studied age range, exposure could never have resulted in increased odds ratios if MP use exerted an adverse effect. Neither if MP use would have induced brain tumors (because this would be tumors diagnosed outside the covered age range) nor if MP use increased growth rate of an already induced brain tumor could elevated odds ratios have been obtained.

**Figure 7:**
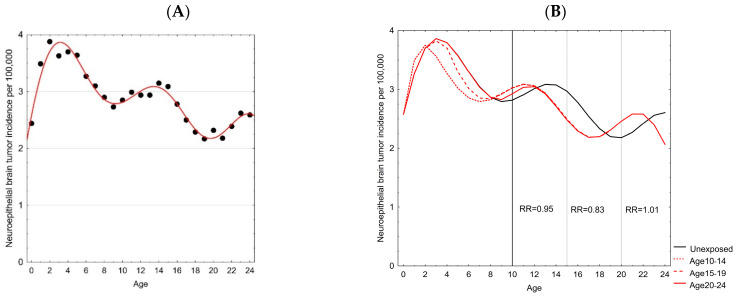
(**A**). Incidence rate of neuroepithelial brain tumors by age and fitted function (data from Central Brain Tumor Registry of the United States, CBTRUS, courtesy of Ms Carol Kruchko). (**B**). Age-incidence function of neuroepithelial brain tumors in unexposed and in those exposed ≥10 years within the age groups assuming an increased tumor growth rate from exposure to wireless phones resulting in a shift in the incidence by x/5 years (x = duration of exposure). Incidence rate ratios (RR) from these shifts within age groups are shown computed as the geometric mean of the ratios of shifted (red) to the original (black) incidence functions over the age-years within each age group.

Concerning the different types of neuroepithelial brain tumors, it turned out that for the age range 0 to 24 years, three functions of type Equation (3) and a constant (1.2 per 100,000) were sufficient to give an excellent fit (R^2^ = 0.96) to the observed age-incidence relationship (Figure 7A and Supplement 2, Table S2.4).

In the MOBIkids study data were reported and risk estimates calculated for three age groups: 10 to 14, 15 to 19, and 20 to 24 years of age. The usage data depicted in Figure S2.5 reflect the secular trend to younger age at onset of use. For determining the consequences of wireless phone use on relative risk estimates, we calculated the expected incidences for ≥10 years of use by arbitrarily modeling a shift by 20% of usage duration. The incidences over the five years span of the age groups in the MOBIkids study were obtained by taking the geometric mean.

Despite the assumption of an increased growth rate from wireless phone use and a shift of ≥2 years of the age-incidence function due to an earlier diagnosis, the resulting relative risk estimates are generally below 1 and only in the highest age group the values are slightly above 1 reflecting the slight increase in the incidences from age 20 onwards (Figure 7B). These results are in line with those reported for MOBIkids [62]. In Table 2 the main results from MOBIkids are shown for the three age groups and the indicators of duration and intensity of use. The results of the calculations assuming a shift corresponding to 20% of MP usage duration are highlighted in this table.

Although most results were statistically not significant, significantly reduced odds ratios and significant (decreasing) trends with increasing duration and intensity of use were only found in the middle age group (15–19 years) consistent with the more pronounced falling trend of the age-incidence function within this age range.

### 3.3. MP Use and Malignant Brain Tumors in the Million Women Study

“During 14 years follow-up of 776 156 women who completed the 2001 questionnaire, a total of 3268 incident brain tumors were registered. Adjusted relative risks for ever vs. never cellular telephone use were 0.97 (95% confidence interval = 0.90 to 1.04) for all brain tumors, 0.89 (95% confidence interval = 0.80 to 0.99) for glioma” [64]. It was concluded that “Our findings support the accumulating evidence that cellular telephone use under usual conditions does not increase brain tumor incidence”. While this is probably a correct statement, it does not imply that cellular telephone use poses no risk for the development of brain tumors.

**Figure 8:**
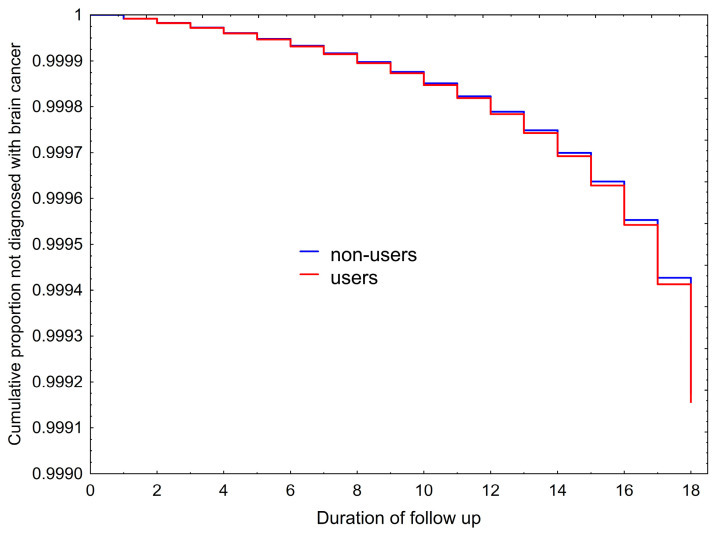
Estimated cumulative proportion not diagnosed with a brain cancer during follow up for non-users and users of a mobile phone under the assumption the incidence at age A is shifted by 32% of the duration of mobile phone use (as obtained from case–control studies in adults) applying Equation (4) Analysis based on the female brain cancer incidence in the UK (averaged over 1990 to 2017) and estimating start year of MP use based on data reported in the update of the Million Women Study (MWS) [64]. The hazard ratio for MP use was 0.98 and statistically not significant pointing to the incapability of studies like the MWS to capture an adverse effect of MP use even if it exists.

The cumulative fractions not diagnosed with a brain tumor calculated according to Equation (4) for users and non-users of a MP are shown in Figure 8. The hazard ratio of mobile phone use was estimated as 0.98 (95% CI: 0.90 to 1.06). The hazard ratio for glioma reported by Schüz et al. [64] was 0.89 (0.80 to 0.99), hence not only significantly below 1 but with a confidence interval well overlapping with the one estimated by us assuming an impact of MP use on tumor growth resulting in a shift in the age-incidence function amounting to 32% of the MP usage duration. Obviously, the Million Women Study will not find a risk estimate above one even if there is a substantial adverse effect as assumed in our analysis.

**Figure 9:**
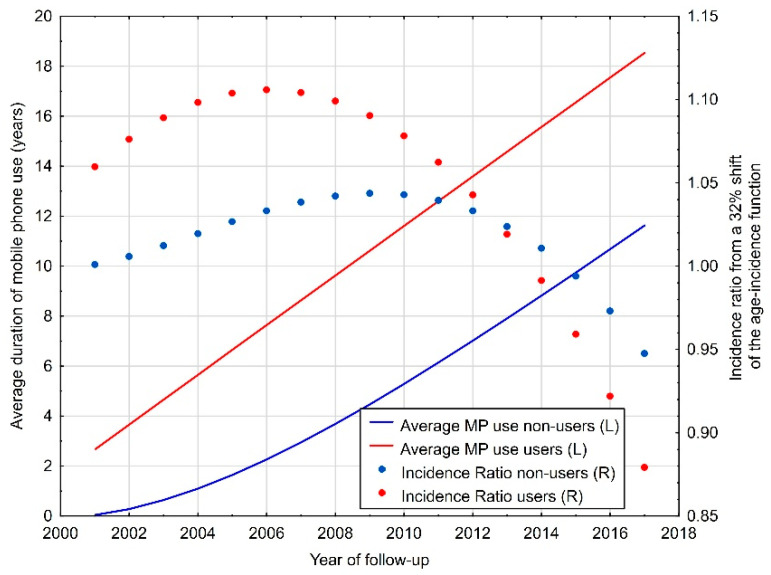
Estimated average mobile phone (MP) usage duration (years) in the cohorts of (initial) users and non-users of the Million Women Study [64] (left axis) and the corresponding impact on the incidence ratio from a 32% shift in the age-incidence function (right axis).

The problem is illustrated in Figure 9. The average duration of MP use is of course increasing in users as well as non-users at study entry (around 2001). While both groups accumulate a substantial duration of MP use, the impact on the magnitude of the shift has to be considered that results increasingly in an incidence rate below one as the age-incidence function is declining. Hence, both groups (initially assigned to user and non-user cohorts) have periods with estimated incidence ratios above one, but as the cohort becomes older the incidence ratios from an assumed shift of 32% of the duration of MP use are below one and stronger so in the (initial) user cohort.

## 4. Discussion and Conclusions

IARC has based the decision to classify radiofrequency electromagnetic fields as a possible human carcinogen (group 2B) mainly on epidemiological studies, in particular the INTERPHONE study and studies by the Swedish group of Lennart Hardell. In a meta-analysis of all case–control studies that included results of long-term MP use we found a significantly elevated odds ratio of 1.22.

What does this mean? The standard interpretation identifies this as an indication of risk while relative risk estimates below 1 are interpreted as an indication of protection. But this is not the only possible explanation. Considering that adult gliomas have average latencies exceeding 20 years [9,11,24], in most cases included in the epidemiological studies the tumor must have been covertly present when cases in these studies started using a MP.

What then does an increased odds ratio mean? We have shown that this is consistent with a shift in the age-incidence function by 32% of the MP usage duration. In other words, assuming that the adverse effect of MP use consists in increasing the growth rate of an already covertly present malignancy and, consequently, earlier diagnosis is, in adults, consistent with the increased odds ratios found in epidemiological studies. This is due to the positive slope of the age-incidence function between 20 and about 70 years of age.

Applying the same rationale to the MOBIkids study of neuroepithelial brain tumors in the age range 10 to 24 years leads to relative risks estimates that are below 1 in the younger age groups and slightly above 1 in the oldest age group of 20 to 24 years. This is qualitatively in line with the risk estimates reported for these age groups that were below 1 in the younger age groups and slightly above 1 in the oldest age group. Which is, according to our interpretation, due to the predominantly declining trend of neuroepithelial brain tumors from the early years of life until about 20 years of age. The observation that statistically significant odds ratios below 1 and significantly decreasing risk estimates with increasing duration and intensity of use occurred only in the middle age group of 15 to 19 years when the declining incidence trend is most pronounced, strongly supports our hypothesis.

While the Million Women Study has been interpreted as generally showing no indication of an increased risk of MP use, we have demonstrated that assuming brain tumors were already covertly growing when the participating peri-menopausal women started MP use will generally result in risk estimates with hazard ratios close to one and, in fact, more likely below 1 because the age-incidence function culminates during follow-up and strongly declines thereafter. The stronger the adverse effect of MP use and consequently the higher the shift, the further below 1 would be the risk estimate. We have shown that already a shift of 32% of the MP usage duration results in a hazard ratio below 1.

It is worthwhile to mention that this interpretation is not only consistent with the epidemiological findings of MP use and brain tumors but can be extended even to findings of a large randomized controlled trial as shown in Supplement 1 for hormone replacement therapy (HRT) and breast cancer. All findings of the Women’s Health Initiative study concerning the adverse effects of HRT are consistent with the assumption of an effect of HRT on latent breast tumor growth. This is in line with considerations about the mechanism of action since estrogen is a mitogen, and hormone-receptor-positive breast tumors would grow faster if higher levels of estrogen are present in the tissue.

Already Sir Austin Bradford Hill [70] had pointed to the difficulties when considering the temporal relationship between an exposure and a disease. For chronic diseases like cancer that remain unnoticed for a long period of time, establishing a clear sequence of events is difficult or even impossible. For new technologies to which individuals are only exposed for short durations compared to the natural history of the disease, an agent may rather be adverse by affecting disease progress. We have hypothesized that there are three theoretical possibilities of such adverse influences: an effect on disease severity by shifting the disease process towards higher malignant features, re-initiating disease development in dormant or regressive disease, and increasing the disease process thereby leading to earlier diagnosis. Focusing on the latter effect, we have shown that all epidemiological findings on the relationship between MP use and brain cancer can be reproduced by shifting the age-incidence function by a certain proportion of the duration of MP use.

It is important to note that this feature does not depend on the type of study. From randomized controlled trials and cohort studies to case–control studies, results will always depend on the age-incidence function if the agent under investigation acts by increasing tumor growth rate. In principle, there are study designs that directly investigate the presence of such a growth-promoting activity. For example, in individuals dying from causes unrelated to brain cancer, autopsies can reveal whether they had an undiagnosed brain tumor. The volume of this tumor could be related to the duration of MP use. However, such a study, given the rarity of brain tumors, is not really feasible. Often, when facing difficulties to study an adverse effect in humans, animal experiments could provide some insight. It can be pointed out that animal studies of exposure to MP frequencies using chemical or habitual tumor initiation found an increased risk, e.g., [71,72,73]. But also animal experiments that are done in ordinary laboratory animals without a tumor-initiating paradigm [74,75] can be interpreted in the same sense since all animals develop tumors spontaneously. If an exposure increases their multiplicity or incidence this can be due to an effect on tumor growth as well. Investigating the etiology and pathophysiology of a malignancy in the context of establishing the mechanism of action of an agent is a long and difficult endeavor and there are no shortcuts in this procedure.

Although in the past decades many in vivo and in vitro mechanistic studies have been performed, they focused on a hypothesized genotoxicity of radiofrequency and microwave fields. Nevertheless, there are some studies that support the hypothesis of a tumor-growth-favoring activity of such exposures, e.g., Volkow et al. [76] by applying glucose-uptake positron-emission tomography showed an increase in local cerebral metabolism after exposure to a mobile phone. There are other studies summarized in the IARC monograph [41] assessing gene and protein expression in exposed tissues that indicate an increased activity of external-signal regulated kinases in exposed tissues, which, among other effects, would increase proliferation. We have shown recently that experimental exposure of volunteers for five days (1950 MHz UMTS for 2 h/day) resulted at a SAR of 1.6 W/kg in a disturbance of the cell cycle and cytotoxic effects in exfoliated buccal mucosa cells [77]. Such effects may under chronic exposure conditions lead to advantages for tumor cells over normal cells due to the resistance of the former against apoptosis. Also, other widely recognized effects of MP radiation in vitro or ex vivo such as induction of oxidative stress and initiation of anti-oxidative defense mechanisms [78] could affect tumor growth. However, it needs to be emphasized that further evidence based on investigations focusing specifically on growth of deviant cell populations is needed.

To validate our interpretation, also other evidence could be relevant. If the age-incidence function is shifted to younger age, brain tumor cases with prior long-term MP use should be younger at diagnosis than non-users. The shift could also affect case fatality rates, as diagnosis would occur at a younger age and age at diagnosis (given the brain cancer type) is the most important predictor of survival. Effects on tumor growth could also impact distribution of tumor grades as increased growth could broaden this distribution (e.g., secondary glioblastoma multiforme would be less frequent since the primary tumor would already become symptomatic, while other types may faster progress to higher malignant stages).

The standard interpretation of a risk estimate associated with a relative risk above 1 is that of an increased risk of the disease in question and an estimate below 1 that of a protecting activity against the disease. This standard interpretation has to be questioned in situations when the agent is acting on an already ongoing disease process. Our argument that in this case the shape of the age-incidence function in the relevant age range is crucial makes the standard interpretation inapplicable. If the slope of this function is positive an agent that is adverse by accelerating the disease process will result in a risk estimate above 1. However, if the slope of this function is negative, an agent, which is adverse by accelerating tumor growth, will have risk estimates below 1. Hence it is evident that the relative risk as such cannot support an assessment of adversity or protectiveness in every situation. Even in the case a relative risk estimate is 1 this can still be consistent with the activity of an adverse agent.

Since the interpretation depends on the history of the disease one has to be cautious in the interpretation of relative risk estimates from epidemiological studies that are in individuals whose disease was already ongoing when exposure commenced, because the standard interpretation may not always be the correct one.

Concerning MP use, some have put forward the argument that given the sharp rise in the number of users between mid-1990s and ~2005, increased incidences of brain cancers should have been observed as well, e.g., [79,80,81,82]. However, this expectation would only be justified if the relative risk estimates from analytical epidemiological studies are interpreted in the standard manner. Since this standard interpretation does not apply if an agent commences after the disease development started, expectations must be reconsidered. Applying the same rationale as for analytical epidemiological findings reveals that any increase in incidence would remain within the annual fluctuations of incidences in all age groups. Even if disregarding the huge differences in exposure patterns and intensities of analog and early digital MPs that have been assessed in the studies reported above compared to later MP generations and the increase in the usage of hands-free devices, no noticeable increase in incidences can occur given our interpretation of relative risks.

This aspect has also to be considered when interpreting the epidemiological evidence utilized in our analyses. The data obtained in all these studies refer to MP technologies that are no longer used. Evidence points to a moderately increased risk for brain tumors of analog and early digital phones. For later MP generations and especially for smart phones that have been marketed from about 2007 onwards there is no evidence at all since no study included subjects with long-term use of this technology.

Another caveat refers to the only moderately increased risk in adults that is however consistent with a substantial effect on tumor growth rate. While more pronounced relative risk estimates cannot be expected, the potential of biases and confounding related to the meta-analytical risk estimates reported here have to be taken into consideration (a more comprehensive discussion can be found in Supplement 2).

It has to be borne in mind that our results are derived from a modeling approach. First, models can only be as good as the data base they are built on. Not only the data from analytical epidemiological studies have their limitations and could be subject to bias and confounding, but also descriptive epidemiology arising from registry information is potentially biased due to incomplete reporting, allocation bias into diagnostic categories and instable denominators in regions with substantial population fluctuations. Second, our hypothesis is currently mainly tested using indirect approaches. While the possibility of direct tests within epidemiology has been addressed and found difficult and hardly feasible, tests applying in vitro, ex vivo, as well as long-term animal experiments are possible and recommended.

While some may consider interpretation of epidemiological evidence of only academic interest, it is indeed of eminent public health importance. The opinion that cancer inducing agents are of greater relevance than those that ‘merely’ have an impact on already induced tumors is highly questionable. Rather, at least for most agents, the opposite is true. The reason is that there are vastly more covertly existing malignant clones than those growing to a cancerous lesion that gets diagnosed. Shifting the balance only slightly in favor of malignant development will have a great impact on incidence. Concerning mobile phone use, our interpretation that exposure (at least for the types of phones for which epidemiological evidence is available) leads to an increase in tumor growth rate has consequences on public health policy and on research as well. Since a growth-promoting activity can only operate as long as vulnerable tissue is exposed; usage recommendations must focus on reducing duration and intensity of exposure to such tissues. Also, the practice to assess SAR in any contiguous tissue should be reconsidered as only vulnerable tissues are of relevance (i.e., bone marrow, meninges and brain tissue). Concerning research, in our opinion the likely fruitless focus on genotoxicity of radiofrequency and microwave fields should be abandoned and more research should concentrate on cellular growth and intracellular signaling features. Very likely a growth-promoting effect has a threshold and establishing such a threshold would provide a basis for the derivation of guideline levels.

## Figures and Tables

**Table 1:** Synopsis of case–control studies of mobile phone (MP) use and glioma risk entering the meta-analysis.

Study	Period	Region	Age Range	Duration of Mobile Phone Use	# Cases	# Controls	Comments
[27]	2004–2006	France	≥16	≥10 y	22	31	
[65]	1997–2003/ 2007–2009	Sweden	20–80/18–75	>10–15 y	71	113	Analog MP
					189	471	Digital MP
					211	476	Total
[22]	2000–2004	13 countries	30–59	≥10 y	252	232	
					190	150	Appendix 2 of [22]
[66]	2002–2007	Korea	15–69	>7 y	100	108	

# Number of…

**Table 2:** Main results of the MOBIKids study [62]. Odds ratios (and associated *p*-values) for neuroepithelial brain tumors in relation to time since start of wireless phone use by age category. Analyses adjusted for parental education. Highlighted in blue background color are the estimates of relative risks from a shift in the age-incidence function by 20% of the mobile phone usage duration. Statistically significant results are indicated in bold.

Age Group	10–14 Years		15–19 Years		20–24 Years	
	OR (*p*-Value)	95% CI	OR (*p*-Value)	95% CI	OR (*p*-Value)	95% CI
***Regular wireless phone user 1 year before diagnosis***						
No	1		1		1	
Yes	0.87 (0.447)	0.61–1.25	0.62 (0.276)	0.26–1.45	1.06 (0.947)	0.19–5.83
***Time since start of use of wireless phones (years)***						
<1 year	1		1		1	
1–4 years	0.82 (0.319)	0.55–1.20	0.79 (0.608)	0.32–1.94	1.76 (0.338)	0.25–12.53
5–9 years	0.98 (0.924)	0.65–1.49	0.55 (0.184)	0.23–1.34	0.97 (0.973)	0.17–5.43
10+ years	0.82 (0.544)	0.43–1.55	0.45 (0.088)	0.18–1.13	1.02 (0.982)	0.18–5.81
*p*-value. linear trend test	0.85		**0.02**		0.85	
Estimate from shift in the age-incidence function	0.95		0.83		1.01	
***Age specific quintiles of cumulative number of calls with wireless phones ^1^***						
NRU.1st_Q	1		1		1	
2nd_Q	1.04 (0.856)	0.68–1.59	0.94 (0.807)	0.57–1.54	1.55 (0.139)	0.87–2.78
3rd_Q	0.72 (0.170)	0.45–1.15	0.62 (0.072)	0.37–1.05	0.87 (0.666)	0.46–1.63
4th_Q	0.88 (0.578)	0.56–1.38	0.78 (0.348)	0.46–1.30	0.92 (0.795)	0.49–1.72
5th_Q	1.09 (0.727)	0.67–1.76	**0.47** (0.012)	0.26–0.84	1.15 (0.664)	0.61–2.15
*p*-value. linear trend test	0.81		**0.01**		0.74	
***Age specific quintiles of cumulative call time with wireless phones (hours) ^2^***						
NRU.1st_Q	1		1		1	
2nd_Q	1.04 (0.854)	0.68–1.57	0.79 (0.354)	0.48–1.30	0.77 (0.380)	0.43–1.38
3rd_Q	0.76 (0.245)	0.48–1.21	0.67 (0.140)	0.39–1.13	0.7 (0.207)	0.40–1.21
4th_Q	0.9 (0.645)	0.58–1.42	**0.52** (0.021)	0.30–0.91	0.57 (0.059)	0.32–1.03
5th_Q	0.84 (0.461)	0.53–1.34	0.61 (0.068)	0.36–1.04	0.65 (0.152)	0.36–1.17
*p*-value. linear trend test	0.36		**0.03**		0.07	

^1^ Age specific quintiles for cumulative number of calls (Q1 to Q5): Age 10–14 years: <282; 282-<750; 750-<1620; 1620-<3891; ≥3891. Age 15–19 years: <1103; 1103-<2738; 2738-<5076.8; 5076.8-<10802; ≥10802. Age 20–24: <2959; 2950-<6582; 6582-<11340; 11340-<19876; ≥19876. ^2^ Age specific quintiles for cumulative call time (hours) (Q1 to Q5): Age 10–14 years: <10.5; 10.5-<36, 36-<82; 82-<220.7; ≥220.7. Age 15–19 years: <47.6; 47.6-<142.7; 142.7-<361; 361-<850; ≥850; Age 20–24 years: <216; 216-<492; 492-<1022; 1022–2303; ≥2303. NRU—not regular use.

## Data Availability

All data used for this study are publicly available (with links provided in the Reference section) or are contained in the Supplementary Files.

## Supplementary Materials

The following supporting information can be downloaded at: https://www.mdpi.com/article/10.3390/epidemiologia7030086/s1, There are two supplementary files: Supplement 1: “Hormone Replacement Therapy and Breast Cancer Risk” containing application of the concept presented in the main text to hormone replacement therapy and breast cancer; Supplement 2 containing further details expanding the main text with the sections: “Considerations about biases in case-control studies” and “Supplemental information about the estimation procedure”.
