# Supplementary material for: Interpretation of Epidemiological Studies on the Relationship Between Mobile Phone Use and Cancer
Source: Epidemiologia (Basel). 2026 Jun 17;7(3):86. doi: 10.3390/epidemiologia7030086 (PMC13298853; doi:10.3390/epidemiologia7030086)
Supplement: Supplementary file 1 [file epidemiologia-07-00086-s001.zip › Epidemiologia Interpretation Supplement 1 without Endnote.pdf]

Supplement 1 of “Interpretation of Epidemiological Studies on the Relationship Between Mobile Phone Use and Cancer” – Hormone Replacement Therapy and Breast Cancer Risk

Michael Kundi[1*] and Hans-Peter Hutter[1]

[1]Medical University Vienna, Center for Public Health, Institute for Environmental Health, Kinderspitalgasse 15, Vienna, Austria

*Correspondence to:

Prof.Dr.Michael Kundi

Medical University Vienna, Center for Public Health, Institute for Environmental Health, Kinderspitalgasse 15, Vienna, Austria

michael.kundi@meduniwien.ac.at

Tel: +43-1-40160-34900

Fax: +43-1-40160-924903

When hormone replacement therapy (HRT) was reassessed by the International Agency for Research on Cancer (IARC), evidence about carcinogenicity in humans from epidemiological studies and randomized controlled trials (RCTs) was already sufficient to classify combined estrogen-progestogen HRT as a group I carcinogen (carcinogenic in humans) [1], although evidence in experimental animals was limited and suggesting an effect on tumor latency only. In the case of HRT, since estrogens are mitogens, it is straightforward to assume HRT acts adversely by increasing tumor growth rate and thereby leading to earlier diagnosis.

In order to show that results of HRT studies can be reproduced by the assumption of an effect on tumor growth only, we modelled breast cancer incidence I_a at age a as a function of previous DNA lesions (that are considered the starting point of the malignant process) and the latency distribution beginning with this event. It is assumed that with probability p_t such an event occurs at age t and that with probability $\pi(a\text{-}t \leq L < a\text{-}t\text{+}1)$ this will lead to diagnosis at age a conditional on surviving until age a. Latency distribution is modelled as a Weibull distribution with parameters τ and b. Since below age 8, breast tissue is not yet developed in most women, analysis starts at age 8.

$$I_a = \sum_{t=8}^{a} p_t \cdot \pi(a - t \leq L < a - t + 1) \cdot (1 - S_t + S_a) \qquad \text{(Eq.1)}$$

Where S_t and S_a are the survival probabilities at age t and a, respectively.

p_t is assumed to increase from zero below age 8 to p_B at age 20 and remains constant thereafter until menopause where it changes from age 41 to 60 to reach p_{PM}, the post-menopausal value, at age 60 and remains constant after this age.

Modelling latency L as a Weibull distribution leads to the following expression (2).

$$\pi(a - t \leq L < a - t + 1) = e^{-\left(\frac{a-t}{\tau_t}\right)^{b_t}} - e^{-\left(\frac{a-t+1}{\tau_t}\right)^{b_t}} \qquad \text{(Eq.2)}$$

The Weibull parameters τ and b were allowed to vary with age t at onset of a malignant process, however, it turned out that b_t=b for all t. For the parameter τ the following variation was chosen:

$$\tau_t = f(t) \cdot \tau \quad f(t) = \begin{cases} t \leq 60 & e^{-\frac{t-8}{85}} \\ t > 60 & 0.5 \end{cases} \qquad \text{(Eq.3)}$$

Hence τ decreases from age 8 to 60 when it reaches 54% of the initial value and remains at 50% thereafter.

For the estimation of parameters p_B, p_{PM}, τ, and b the Levenberg-Marquard algorithm has been used on the US breast cancer incidence data (SEER 2014-18) as basis (Fig.S1.1). Except for age above 74 years, the estimates are also close to the rates reported for 1991-95. Hence, for the purpose of assessing the impact of an agent that increases tumor growth rate the obtained estimates are sufficient.

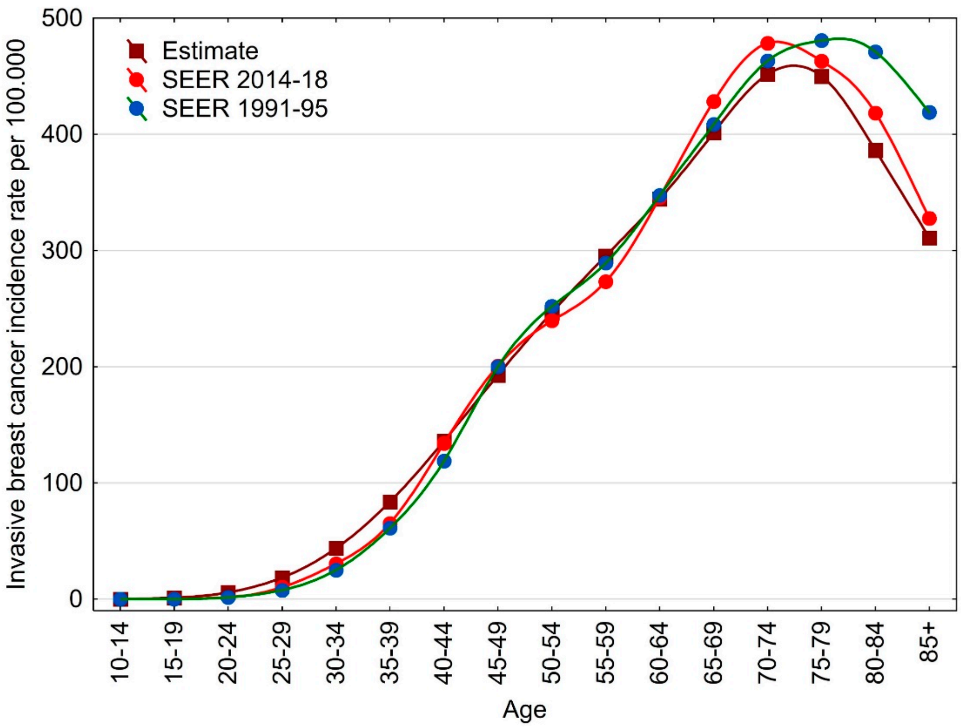

Figure S1.1. Incidence of invasive breast cancer per 100.000 women in the USA (2014-18) and fitted model (Eq.1). For comparison, also incidences for the years 1991-95 are shown.

Note that for estimating breast cancer incidence, no other information about the population as overall survival was required.

The parameter estimates were: p_B=0.0023, p_{PM}=0.0054, τ=36.1, and b=3.15. According to these estimates approximately 230 of 100.000 women experience a malignant transformation every year between age 20 and 40, this value increases thereafter to 540 of 100.000 women until age 60 and remains at that level thereafter (and may decline at very old age because this was not a specific target of the estimation). Estimated distribution of latencies are shown for different age in Fig.S1.2.

Consistent with estimates from the literature [2-4] average latencies vary between about 20 and 30 years. Latency decreases with age, maybe due to a depletion of stem cells. Apparently, a significant number of tumors are not diagnosed during lifetime and may remain clinically inconspicuous for the future life after initiation.

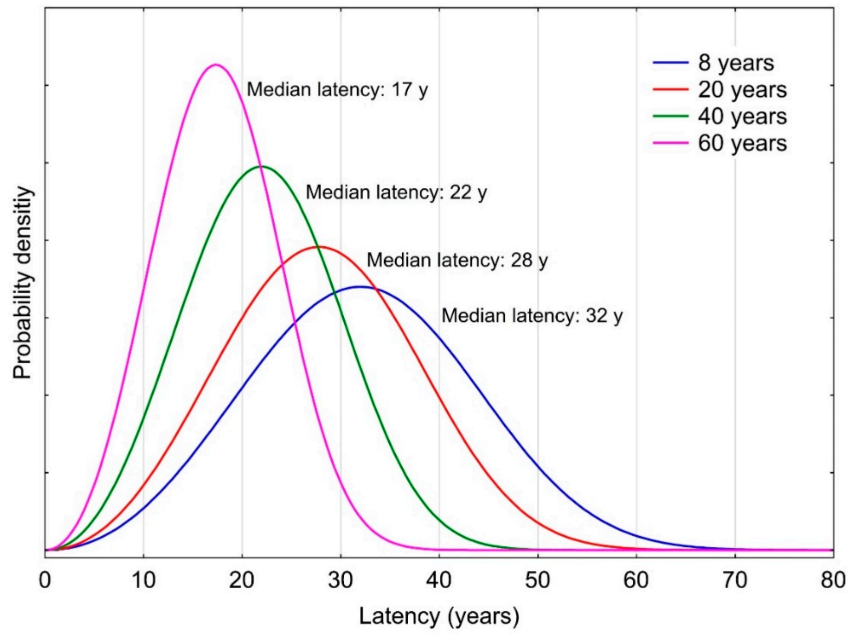

Figure S1.2. Estimated latency distribution for different age at malignant transformation

Although tumor sizes at diagnosis are decreasing during the last years as a consequence of improvements in mammography techniques and increased screening rates, an average tumor size of 2 cm can be assumed for the period when hormone replacement therapy (HRT) was studied in relation to breast cancer risk. Such a tumor will harbor around 30 to 40 million cells. Thus, we can expect around 25 generations from the initial deviant cell clone to diagnosis. Hence, average generation time will be about one year.

The overall hazard ratio from RCTs summarized in the IARC assessment was 1.24 (95% CI: 1.09-1.42). We can apply our model (Eq.1) to compute incidence ratios from a shift of latencies. The age range in the Women's Health Initiative (WHI) study [5] was 50-79 years with an average of 64 years. Within this age-range, incidences are increasing almost linearly (Fig.S1.1). The expected annual incidence within this WHI sample of women receiving a combination HRT is 215 cases per 100,000 (note that for the purpose of this assessment it is not necessary to make a distinction between incidence density and cumulative incidence because incidences were virtually constant over the study periods in this age range). In order to get an incidence ratio of 1.24, in HRT users 267 cases per 100,000 must occur in the age range 56-85 (6 years later since the average follow-up was 5.6 years). Modelling a reduction of latency by 4 years gives an incidence ratio of 1.24. The age incidence functions are shown in Fig.S1.3.

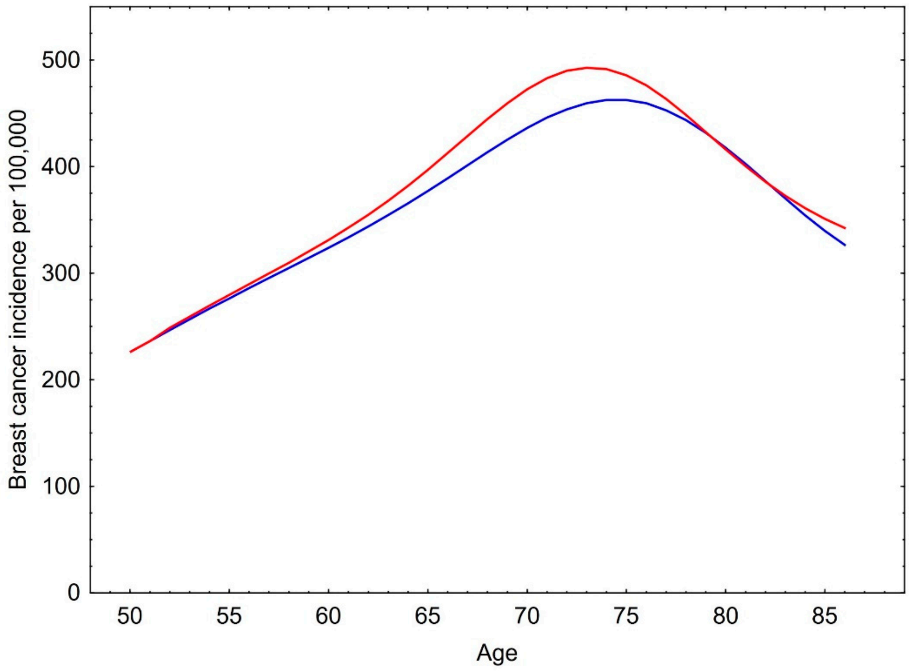

Figure S1.3. Age incidence function observed (blue) and expected from a shift in latency by 4 years (red) for those beginning to use hormone replacement therapy between age 50 and 79 (corresponding to the WHI study). Average incidence ratio: 1.24.

In order to get an impression of the predicted dynamics of the breast cancer incidence development, instead of a start of HRT between 50 and 79 years, start was modelled at age 50. From Fig. S1.4 it is obvious that the incidence curves start to diverge at about 4 years after the onset at age 50, consistent with the observation of the WHI trial.

It can be concluded that all observations surrounding breast cancer risk associated with HRT can be reproduced by assuming that the impact of such an intervention on breast cancer was restricted to an effect on latency. Due to the long latencies of breast cancer, it can be assumed that onset of HRT in those later diagnosed with breast cancer was predominantly or totally in those that had already a latent tumor. Therapy led to an increased growth rate and, consequently, to an earlier diagnosis. Total number of tumors may only increase due to diagnosis before occurrence of death from competing causes, since no increase in rate of initiation is necessary to explain the increased incidence.

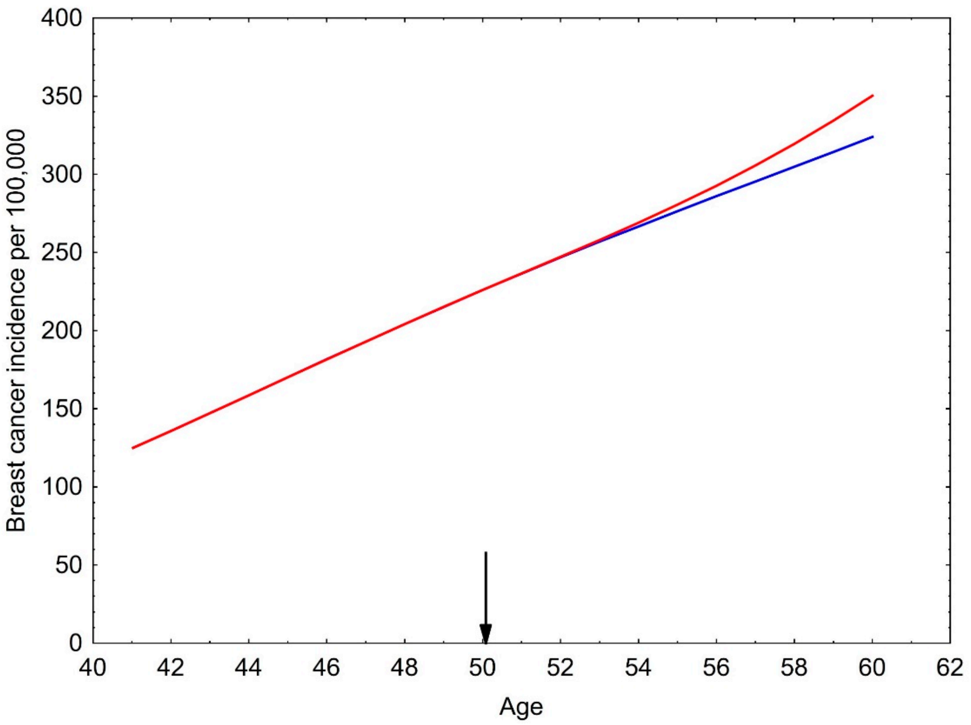

Figure S1.4. Age-incidence functions under the assumption of no shift (blue) and assuming a shift of the latency distribution by 4 years (red) after onset of hormone replacement therapy assumed to start at age 50.

References

1. IARC. *Working Group on the Evaluation of Carcinogenic Risks to Humans. World Health Organization. International Agency for Research on Cancer. Combined estrogen-progestogen contraceptives and combined estrogen-progestogen menopausal therapy*; World Health Organization: **2007**; Volume 91.
2. Goss, P.E.; Sierra, S. Current perspectives on radiation-induced breast cancer. *Journal of clinical oncology* **1998**, *16*, 338-347.
3. Olsson, H.; Baldetorp, B.; Fernö, M.; Perfekt, R. Relation between the rate of tumour cell proliferation and latency time in radiation associated breast cancer. *BMC cancer* **2003**, *3*, 1-5.
4. Thomas, D. Epidemiologic and related studies of breast cancer etiology. *Reviews in cancer epidemiology* **1980**, *1*, 153-217.
5. Chlebowski, R.T.; Hendrix, S.L.; Langer, R.D.; Stefanick, M.L.; Gass, M.; Lane, D.; Rodabough, R.J.; Gilligan, M.A.; Cyr, M.G.; Thomson, C.A. Influence of estrogen plus progestin on breast cancer and mammography in healthy postmenopausal women: the Women's Health Initiative Randomized Trial. *Jama* **2003**, *289*, 3243-3253.
