# Supplementary material for: Interpretation of Epidemiological Studies on the Relationship Between Mobile Phone Use and Cancer
Source: Epidemiologia (Basel). 2026 Jun 17;7(3):86. doi: 10.3390/epidemiologia7030086 (PMC13298853; doi:10.3390/epidemiologia7030086)
Supplement: Supplementary file 1 [file epidemiologia-07-00086-s001.zip › Epidemiologia Interpretation Supplement 2 without Endnote.pdf]

Supplement 2 of “Interpretation of Epidemiological Studies on the Relationship Between Mobile Phone Use and Cancer”

Michael Kundi[1*] and Hans-Peter Hutter[1]

[1]Medical University Vienna, Center for Public Health, Institute for Environmental Health, Kinderspitalgasse 15, Vienna, Austria

*Correspondence to:

Prof.Dr.Michael Kundi

Medical University Vienna, Center for Public Health, Institute for Environmental Health, Kinderspitalgasse 15, Vienna, Austria

michael.kundi@meduniwien.ac.at

Tel: +43-1-40160-34900

Fax: +43-1-40160-924903

Table S2.1. Selection bias factors for the studies included in the main analysis

Table S2.2. Estimated parameters for adult glioma by age (median latency calculated from Weibull parameters)

Table S2.3. Relative risks associated with various shifts of the age-incidence function by a percentage of the mobile phone usage duration

Table S2.4. Estimated parameters for childhood neuroepithelial brain tumors (median latency calculated from Weibull parameters)

Figure S2.1. DerSimonian & Laird random effects meta-analysis of case-control studies of long-term mobile phone use and glioma risk adjusted for selection bias. Heterogeneity was completely removed (I^2=0% as compared to a highly significant heterogeneity of 74.2% of the unadjusted analysis).

Figure S2.2. Ratio of self-reported to provider obtained cumulative duration of calls [5]

Figure S2.3. Glioma incidence in males and females from the SEER database (1992-2000) and fitted function according to Eq.1

Figure S2.4. Incidence rates of different types of neuroepithelial brain tumors by age (data from CBTRUS, courtesy of Ms Carol Kruchko) – (NOS, not otherwise specified, GBM, glioblastoma multiforme)

Figure S2.5. Proportion of regular wireless phone users by age group in controls of the MOBIKids study

Considerations about biases in case-control studies

In observational studies the most important biases are selection and information bias. Since we are dealing with case-control studies selection bias may result from enrolling cases and/or controls in correlation with the agent under study – in this case MP use. The selection odds ratio (or bias factor) can easily be determined e.g. [1]. It is given by Eq.1, where S_{ED}, $S_{E'D'}$, $S_{E'D}$, and $S_{ED'}$ denote the selection probabilities of exposed (E) cases (D), not exposed (E') controls (D'), not exposed cases and exposed controls, respectively.

$$Bias\ factor = \frac{s_{ED} \cdot s_{E'D'}}{s_{E'D} \cdot s_{ED'}} \qquad \text{Eq.1}$$

It can be seen that whenever the selection fraction either of cases or controls differ with respect to exposure there would be bias unless for both cases and controls the ratio of selection among exposed and non-exposed is equal. In practice, however, the selection fractions are unknown. The bias factor can be estimated if the exposure status of non-participants can be determined or if exposure fraction in the target population is known.

The potential for selection bias increases with decreasing participation rates. Table S2.1 shows participation rates of the studies included in this assessment. From the large differences in participation rates between cases and controls in the Interphone study and some of the other trials it can be concluded that there is a potential for selection bias that needs to be assessed.

The Interphone Study Group obtained some data (from 11 participating countries) of non-participants by contacting them with a nonresponse questionnaire (NRQ) obtaining a few data including regular mobile phone use [2]. For estimating the bias factor there are four subgroups of cases (in the formula first index 1) and controls (first index 0) to be differentiated: interviewed participants (second index 1), refusals with exposure status known (second index 2), refusals with exposure status unknown (second index 3) and other non-participants (second index 4).

$$Bias\ factor = \frac{\rho_{11} + \sum_{j>1} \frac{1-\pi_{1j}}{1-\pi_{11}} \rho_{1j}}{\rho_{11} + \sum_{j>1} \frac{\pi_{1j}}{\pi_{11}} \rho_{1j}} \cdot \frac{\rho_{10} + \sum_{j>1} \frac{\pi_{0j}}{\pi_{01}} \rho_{0j}}{\rho_{10} + \sum_{j>1} \frac{1-\pi_{0j}}{1-\pi_{01}} \rho_{0j}} \qquad \text{Eq.2}$$

Where ρ_{ij} refer to the proportions of participants in the four groups j among cases (i=1) and controls (i=0) and π_{ij} are the rates of regular mobile phone users within these subgroups. Vrijheid et al. [2] considered five scenarios for estimating mobile phone user rates in the two subgroups (3 and 4) without information on mobile phone use. Considering the huge differences in reasons for non-participation for cases and controls (with cases mainly not participating due to their advanced illness or being already deceased while controls mainly did not participate because they could not be reached or because they refused – maybe often because they saw no point participating in a mobile phone study when they did not have one) we simplified the approach considering only one scenario: for non-

responders without NRQ and other non-participants the usage rate of NRQ respondents apply for cases, while for controls in non-responders without NRQ and other non-participants a 33% lower usage rate than in NRQ respondents apply.

We calculated average usage rates for non-participants with NRQ by inverse variance weighting of the data reported by [2]. This resulted in a bias factor 0.56 for the Interphone study. Unfortunately for the other studies no data about MP use in non-respondents are available. In order to calculate bias factors also for these studies, we used for all non-respondents the same fraction of mobile phone use as computed from the Interphone study: for controls 78.4% of the usage rate in participants and for cases 97.0%. Results are summarized in Table S2.1.

Table S2.1. Selection bias factors for the studies included in the main analysis

	Participation rate		Mobile phone users[1]		Bias factor
Study	Cases	Controls	Cases	Controls	
Hardell et al. (2006)	90%	89%	64%	54%	0.96
INTERPHONE Study Group (2010)	64%	53%	62%	64%	0.56
Hardell et al. (2013)	87%	85%	96%	92%	0.77
Coureau et al. (2014)	66%	45%	56%	54%	0.79
Yoon et al. (2015)	32%	44%	84%	86%	0.58

[1] among participants

Applying these bias factors to the results on long-term mobile phone use completely removed the heterogeneity between studies as shown in Figure S2.1.

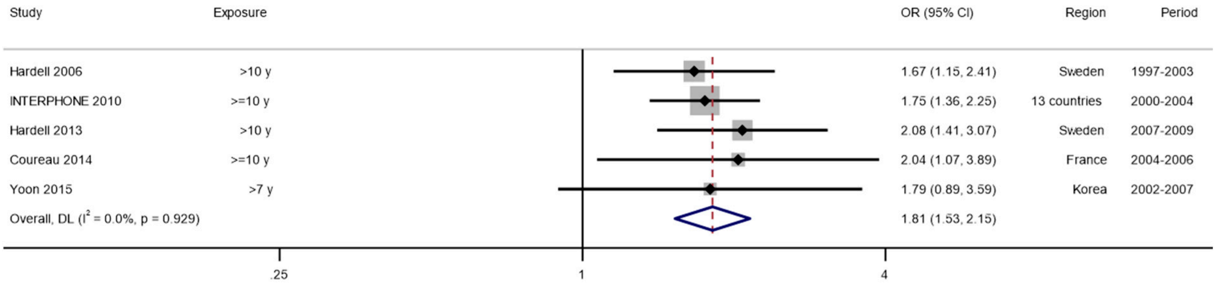

Figure S2.1. DerSimonian & Laird random effects meta-analysis of case-control studies of long-term mobile phone use and glioma risk adjusted for selection bias. Heterogeneity was completely removed (I^2=0% as compared to a highly significant heterogeneity of 74.2% of the unadjusted analysis).

Besides selection bias, information bias is a possible source of distortion of outcomes. In case-control studies with exposure determined from self-reports this bias is specified as recall bias. Some validation studies concerning recall of mobile phone use have been conduction by the Interphone study group either using network provider records or software-modified mobile phones e.g. [3-5]. While there is only a tendency for underestimation of the number of calls and overestimation of duration of calls, the rather large random error needs to be considered. However, it has to be borne in mind that provider

data do not accurately specify exposure either. There are a number of sources of bias in datasets obtained from network operators:

- Some operators only have data on outgoing calls (e.g. data from Australia in [5])
- Calls are registered that although they are only from a non-availability response automatically generated by the mobile phone without actually reaching the called person and without resulting in an exposure
- Especially in earlier years a mobile phone was sometimes shared with another person
- On the other hand, calls that do not connect but result in exposure are not registered (in GSM phones even the highest one since during the first seconds of connecting to the base station the phone is operated at full power)
- It can also not be determined from provider records whether a hands-free device or speaker mode was used that would result in no relevant exposure of the brain

It has been shown, however, by Vrijheid*, et al.* [6] using a simulation approach that random differential and non-differential recall bias results in an underestimation of a true risk. A further simulation study especially focusing on cumulative duration of calls [7] reported "We found that the scenario simultaneously modeling systematic and random reporting errors produced a J-shaped relationship perfectly compatible with the observed relationship from the main Interphone study…" and concluded that "(s)ome uncertainty remains, but the evidence from the present simulation study shifts the overall assessment to making it less likely that heavy mobile phone use is causally related to an increased glioma risk". However, this J-shaped relationship they claim to have reproduced is totally an artifact of an inappropriate scenario with no systematic error in controls and systematic as well as duration dependent error (underestimation of duration below the average and overestimation above) in cases and additionally a 10% higher random error in cases. That these assumptions are not in line with the validation results of the Interphone study is shown in Figure S2.2.

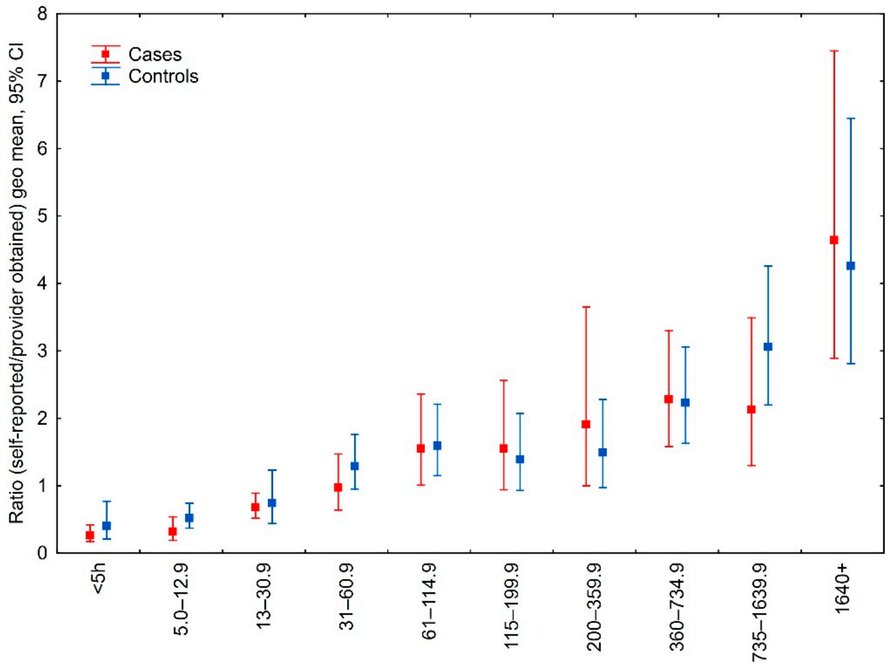

Figure S2.2. Ratio of self-reported to provider obtained cumulative duration of calls [5]

Supplemental information about the estimation procedure

Table S2.2. Estimated parameters for adult glioma by age (median latency calculated from Weibull parameters)

Gender	Age	Probability of transformation[1]	Weibull parameter T	Weibull parameter b	Median latency (years)[2]
Males	0	0.0017689	85.4	6.7	80.9
	10	0.0015682	76.4	3.7	69.2
	20	0.0011496	68.4	3.4	61.3
	30	0.0009489	61.2	3.4	54.9
	40	0.0009120	54.8	3.4	49.1
	50	0.0009068	49.0	3.4	43.9
	60	0.0009060	43.8	3.4	39.3
	70	0.0009060	39.2	3.4	35.2
Females	0	0.0010862	102.6	5.6	96.1
	10	0.0009629	90.6	3.1	80.4
	20	0.0007059	79.9	2.8	70.1
	30	0.0005826	70.5	2.8	61.8
	40	0.0005600	62.2	2.8	54.5
	50	0.0005568	54.9	2.8	48.1
	60	0.0005563	48.5	2.8	42.5
	70	0.0005563	42.8	2.8	37.5

[1] Calculated according to Equ 2 in the main text; [2] Calculated from parameters T and b

Table S2.3. Relative risks associated with various shifts of the age-incidence function by a percentage of the mobile phone usage duration

Shift (% of mobile phone usage duration)	Resulting relative risk
20	1.160
22	1.152
24	1.176
26	1.200
28	1.207
30	1.216
32	1.220
34	1.266
36	1.278
38	1.298
40	1.311

Table S2.4. Estimated parameters for childhood neuroepithelial brain tumors (median latency calculated from Weibull parameters)

Brain tumor type	Probability of transformation	Weibull parameter T	Weibull parameter b	Median latency (years)
1	0.000195	6.3	2.0	5.2
2	0.000165	15.9	4.6	14.7
3	0.000106	25.7	9.2	24.7

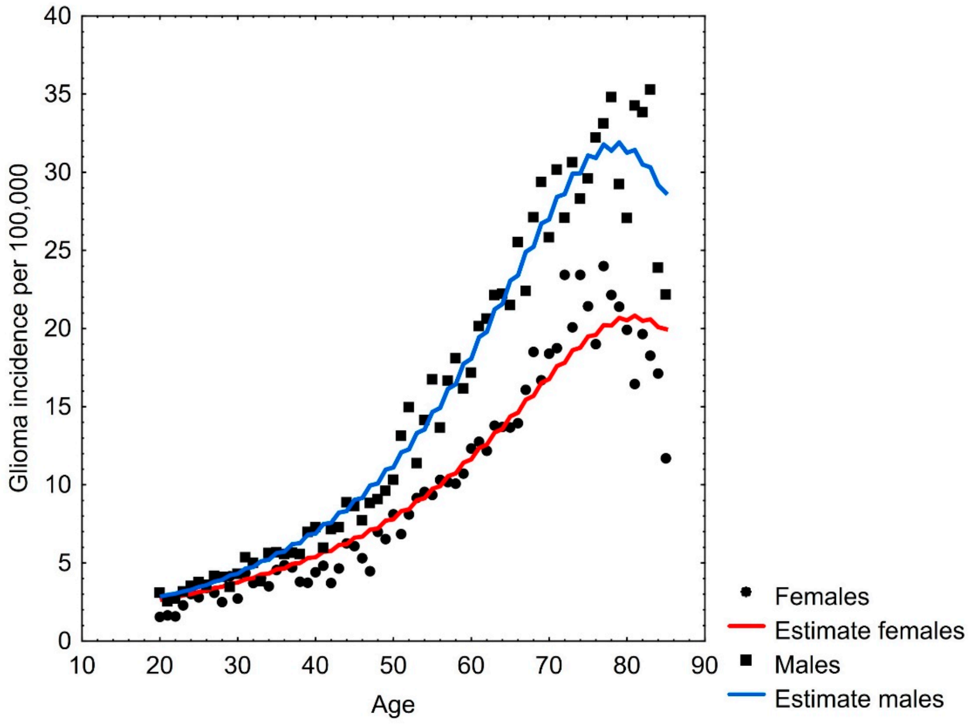

Figure S2.3. Glioma incidence in males and females from the SEER database (1992-2000) and fitted function according to Eq.1

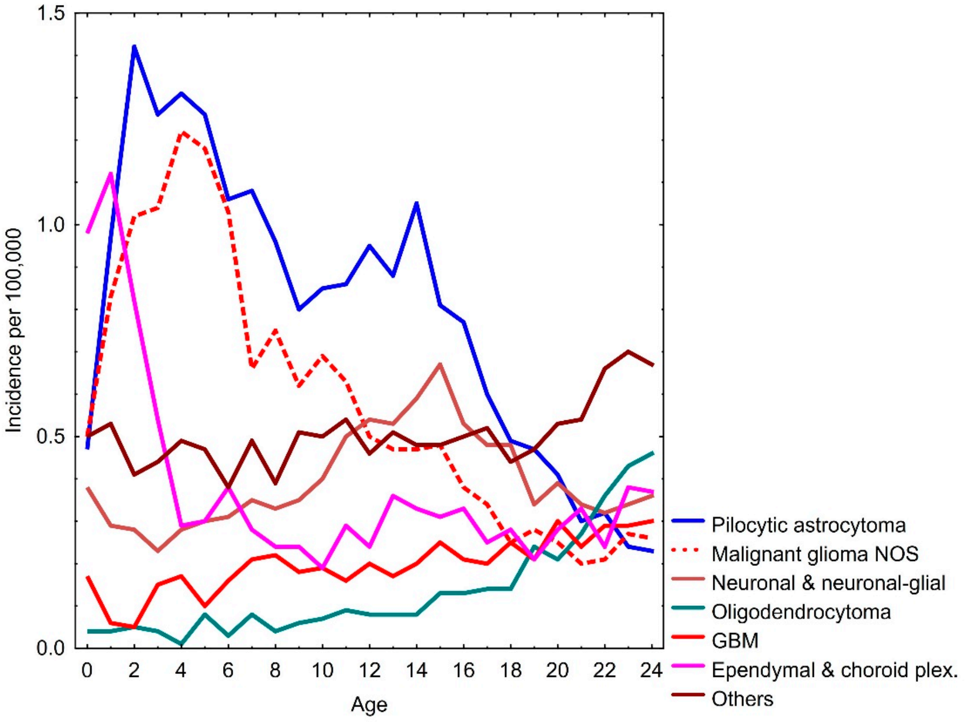

Figure S2.4. Incidence rates of different types of neuroepithelial brain tumors by age (data from CBTRUS, courtesy of Ms Carol Kruchko) – (NOS, not otherwise specified, GBM, glioblastoma multiforme)

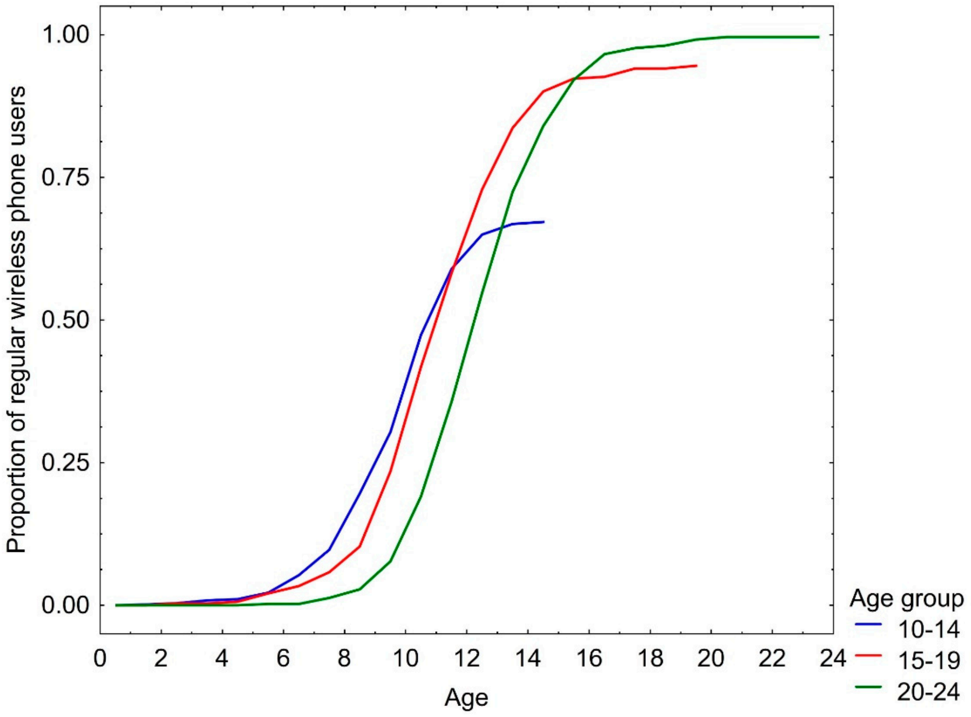

Figure S2.5. Proportion of regular wireless phone users by age group in controls of the MOBIKids study

References

1. Kleinbaum, D.G.; Morgenstern, H.; Kupper, L.L. Selection bias in epidemiologic studies. *Am J Epidemiol* **1981**, *113*, 452-463, doi:10.1093/oxfordjournals.aje.a113113.
2. Vrijheid, M.; Richardson, L.; Armstrong, B.K.; Auvinen, A.; Berg, G.; Carroll, M.; Chetrit, A.; Deltour, I.; Feychting, M.; Giles, G.G.; et al. Quantifying the impact of selection bias caused by nonparticipation in a case-control study of mobile phone use. *Ann Epidemiol* **2009**, *19*, 33-41, doi:10.1016/j.annepidem.2008.10.006.
3. Berg, G.; Schüz, J.; Samkange-Zeeb, F.; Blettner, M. Assessment of radiofrequency exposure from cellular telephone daily use in an epidemiological study: German Validation study of the international case-control study of cancers of the brain--INTERPHONE-Study. *Journal of exposure analysis and environmental epidemiology* **2005**, *15*, 217-224, doi:10.1038/sj.jea.7500390.
4. Samkange-Zeeb, F.; Berg, G.; Blettner, M. Validation of self-reported cellular phone use. *Journal of exposure analysis and environmental epidemiology* **2004**, *14*, 245-248, doi:10.1038/sj.jea.7500321.
5. Vrijheid, M.; Armstrong, B.K.; Bédard, D.; Brown, J.; Deltour, I.; Iavarone, I.; Krewski, D.; Lagorio, S.; Moore, S.; Richardson, L.; et al. Recall bias in the assessment of exposure to mobile phones. *Journal of exposure science & environmental epidemiology* **2009**, *19*, 369-381, doi:10.1038/jes.2008.27.
6. Vrijheid, M.; Deltour, I.; Krewski, D.; Sanchez, M.; Cardis, E. The effects of recall errors and of selection bias in epidemiologic studies of mobile phone use and cancer risk. *Journal of exposure science & environmental epidemiology* **2006**, *16*, 371-384, doi:10.1038/sj.jes.7500509.
7. Bouaoun, L.; Byrnes, G.; Lagorio, S.; Feychting, M.; Abou-Bakre, A.; Béranger, R.; Schüz, J. Effects of Recall and Selection Biases on Modeling Cancer Risk From Mobile Phone Use: Results From a Case-Control Simulation Study. *Epidemiology* **2024**, *35*, 437-446, doi:10.1097/ede.0000000000001749.
